# Synthesis of Au NP@MoS_2_ Quantum Dots Core@Shell Nanocomposites for SERS Bio-Analysis and Label-Free Bio-Imaging

**DOI:** 10.3390/ma10060650

**Published:** 2017-06-13

**Authors:** Xixi Fei, Zhiming Liu, Yuqing Hou, Yi Li, Guangcun Yang, Chengkang Su, Zhen Wang, Huiqing Zhong, Zhengfei Zhuang, Zhouyi Guo

**Affiliations:** MOE Key Laboratory of Laser Life Science & SATCM Third Grade Laboratory of Chinese Medicine and Photonics Technology, College of Biophotonics, South China Normal University, Guangzhou 510631, Guangdong, China; feixixifish@126.com (X.F.); 15692432550@163.com (Y.H.); vaeliyi@163.com (Y.L.); 18819286477@163.com (G.Y.); srrx@163.com (C.S.); Wangz706@126.com (Z.W.); Zhonghq@scnu.edu.cn (H.Z.); 15521027299@163.com (Z.Z.)

**Keywords:** MoS_2_ quantum dots, Au nanoparticles, surface enhanced Raman scattering, tumor cells

## Abstract

In this work, we report a facile method using MoS_2_ quantum dots (QDs) as reducers to directly react with HAuCl_4_ for the synthesis of Au nanoparticle@MoS_2_ quantum dots (Au NP@MoS_2_ QDs) core@shell nanocomposites with an ultrathin shell of ca. 1 nm. The prepared Au NP@MoS_2_ QDs reveal high surface enhanced Raman scattering (SERS) performance regarding sensitivity as well as the satisfactory SERS reproducibility and stability. The limit of detection of the hybrids for crystal violet can reach 0.5 nM with a reasonable linear response range from 0.5 μM to 0.5 nM (R^2^ ≈ 0.974). Furthermore, the near-infrared SERS detection based on Au NP@MoS_2_ QDs in living cells is achieved with distinct Raman signals which are clearly assigned to the various cellular components. Meanwhile, the distinguishable SERS images are acquired from the 4T1 cells with the incubation of Au NP@MoS_2_ QDs. Consequently, the straightforward strategy of using Au NP@MoS_2_ QDs exhibits great potential as a superior SERS substrate for chemical and biological detection as well as bio-imaging.

## 1. Introduction

Transition metal dichalcogenide (TMDC) materials have been intensively studied in recent years for their intriguing properties and unique structures [1,2,3,4]. Molybdenum disulfide (MoS_2_), as a typical TDMC family number, is particularly promising thanks to its superior optical, electronic, and mechanical properties for the wide applications of photocatalysis, hydrogen evolution, and optical sensors [5,6,7,8]. MoS_2_ has a structure built of covalently bonded S-Mo-S single layers interacting by van der Waals forces and each molybdenum atom is surrounded by six sulphur atoms [9]. It is worth noting that, among the various morphologies of MoS_2_, MoS_2_ quantum dots (MoS_2_ QDs) display better optical properties mainly due to their larger direct band gap which directly impacts their optical properties when compared with bulk and monolayer MoS_2_ [10,11,12]. MoS_2_ QDs also exhibit the properties of large surface area, excellent solubility, chemical inertia, high stability, and low toxicity, and are potentially applicable in bio-imaging, fluorescent sensors, and cancer therapies [13,14,15,16,17,18,19]. However, few reports have studied the application of MoS_2_ QDs for surface enhanced Raman scattering (SERS).

SERS is one of the most sensitive and powerful analytical techniques for detecting molecular vibrational fingerprints with high resolution [20]. The typical material for SERS is noble metal (e.g., Au, Ag, and Cu) with nanostructures or rough surfaces which can vastly amplify the SERS signal owing to enhancement mechanism (EM) caused by their local surface plasmon resonance (LSPR) effect [21,22]. In order to obtain better SERS performance, many researchers have made efforts to functionalize noble metal nanoparticles with graphene and its derivatives due to more hot spots being generated and the chemical mechanism (CM) induced by the charge transfer between the SERS substrates and the molecules [23,24,25,26]. Interestingly, we found that the MoS_2_ family can also produce appropriate SERS activity through CM and provide efficient adsorption for a variety of target molecules [27,28]. Moreover, the MoS_2_-Au system can provide strong exciton-plasmon coupling and the coupling of the two resonances are capable of contributing to the SERS enhancement [29,30]. Additionally, sharing a graphene-like structure, MoS_2_ can be produced at a lower temperature which can efficaciously reduce the energy consumption and makes it easier to combine with traditional metal nanoparticles when compared with graphene. Therefore, the MoS_2_-metal nanoparticle composites have advantages of being SERS substrates. Su et al. achieved sensitive SERS detection of rhodamine 6G by employing synthesized Au nanoparticles (Au NPs)-MoS_2_ nanocomposites [31]. Compared with two-dimensional (2D) MoS_2_, 0D MoS_2_ QDs have a larger specific surface area and more edge atoms that can lead to the deficiency of co-ordination of the surface atoms and the increasement of unsaturated bonds for absorbing more analytes, which may realize higher SERS performance [31,32,33].

**Scheme 1:**
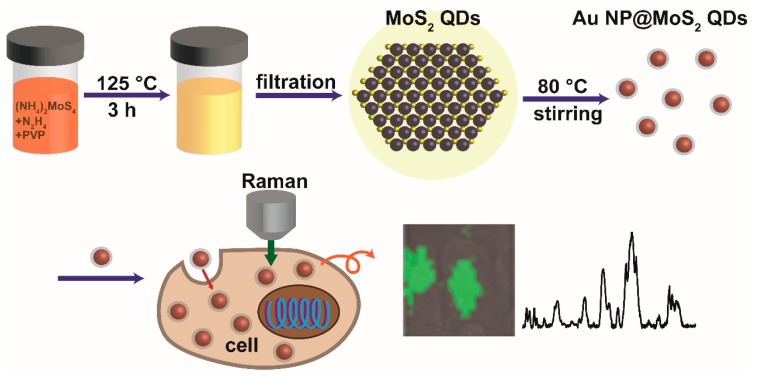
Illustration of the fabrication process of the Au NP@MoS_2_ QDs nanocomposites and their SERS application.

In this work, we report a facile and green method to synthesize Au NP@MoS_2_ QDs nanocomposites with a core@shell nanostructure for the first time. The MoS_2_ QDs, as a reducer, directly react with the HAuCl_4_ in aqueous solution at 80 °C without any other agents (Scheme 1). The pinhole-free, chemical inert, and ultrathin MoS_2_ QDs shell (with a thickness of ca. 1 nm) that meets the basic requirement of shell-isolated nanoparticle-enhanced Raman spectroscopy (SHINERS) can protect the Au-core from the chemical environment, the surface of interest, and the probe molecules [34,35,36]. The as-prepared substrate of AuNP@MoS_2_ QDs nanocomposites makes full use of the synergistic effects of the eigen properties of MoS_2_ QDs and the Au NPs exhibit an admirable SERS effect. Furthermore, the shell-isolated nanoparticles show much better stability than the bare NPs because of the inert coatings [35]. Taking that into consideration with the non-toxic and biocompatible MoS_2_ QDs, we apply AuNP@MoS_2_ QDs as the nanoprobe for the label-free near-infrared (NIR) SERS imaging of 4T1 cells with a satisfactory result, where the Raman features of the cellular compounds have been increased and the intensity of these Raman signals is sharply enhanced. Thus, AuNP@MoS_2_ QDs may be a promising nano-material for applications such as toxicant detection and bio-analysis.

## 2. Materials and Methods

### 2.1. Materials

Ammonium tetrathiomolybdate [(NH_4_)_2_MoS_4_] was purchased from J&K Chemical Ltd. (Shanghai, China). Tetrachloroauric acid (HAuCl_4_), hydrazine hydrate (N_2_H_4_, 80%), and polyvinyl pyrrolidone (PVP, 30 kDa) were bought from China National Medicine Corporation (Shanghai, China). Roswell Park Memorial Institute (RPMI)-1640 and fetal bovine serum (FBS) were obtained from GIBCO (Grand Island, NY, USA). Other reagents were of analytical grade and were used as received without further purification. Deionized water (Milli-Q System, Millipore, Billerica, MA, USA) was used in all experiments.

### 2.2. Preparation of MoS_2_ Quantum Dots

MoS_2_ QDs were synthesized via a facile hydrothermal method with the precursor of (NH_4_)_2_MoS_4_. In detail, 5.5 mg (NH_4_)_2_MoS_4_ was added into a 20 mL PVP solution (1.1 mg/mL) for dissolution through ultrasonication. After complete dissolution, 200 μL N_2_H_4_ (80%), the reducing reagent, was mixed with the solution. Then, the mixture was transferred to a 50 mL Teflon-lined stainless steel autoclave and kept at 125 °C for 3 h. Once the solution was cooled down to room temperature, the impurities were filtered by a 0.22 μm microporous membrane for colation of the unreacted residue. Finally, the solution was concentrated and further purified to remove the PVP and the residual salts by ultrafiltration (M_W_ = 100 k).

### 2.3. Synthesis of Au NP@MoS_2_ QDs Nanocomposites

100 μL of the MoS_2_ QDs solution and 10 mL of ultrapure water were put into a 50 mL conical flask under vigorous stirring. When heated to 80 °C, 200 μL HAuCl_4_ (10 mM) was slowly added drop-wise and the color of the mixture changed to aubergine immediately. After a 10-min reaction, the nanocomposites were collected and centrifuged at 10,000 rpm for 20 min performed three times. The pellet was resuspended in deionized water to obtain the proper concentration. Spherical Au NPs with the same size were also synthesized as the control. The synthesis route was as follows: 0.6 mL of HAuCl_4_ (10 mM) was diluted with 20 mL of ultrapure water under sustained stirring in the heated state. At the time when the solution was boiling, 200 mL of sodium citrate aqueous solution (1%) was added and boiled for another 20 min. The color of the solution changed from golden yellow to jacinth, which indicated the generation of the spherical Au NPs.

### 2.4. Characterization

Transmission electron microscope (TEM) measurements were performed on a Tecnai G2 Spirit T12 (FEI, Hillsboro, OR, USA) operated at 120 kV equipped with an energy-dispersive X-ray (EDX) spectrometer. The UV-Vis absorption spectra of the hybrids were taken by a UV-Vis spectrometer (UV-6100, MAPADA, Shanghai, China). Raman spectra were recorded by using a Renishaw inVia microspectrometer (Renishaw, Derbyshire, England) with an excitation wavelength of 514.5 nm obtained from an Ar^+^ laser.

### 2.5. SERS Experiments

To investigate the SERS effect, the liquid samples for SERS measurements were prepared by a mixture of CV aqueous solutions with different concentrations and the Au NP@MoS_2_ QDs suspension. Then, 4 μL of the mixture was dropped on the Si substrate to perform the instant SERS measurement. The SERS spectra was measured by the Renishaw inVia microspectrometer with a 514.5 nm laser excitation. The laser power on the sample was 1 mW with a 9 s accumulation time. All experiments were independently conducted three times.

For cellular SERS analysis, the precultured mouse mammary cancer (4T1) cells were seeded on quartz substrates, and incubated with Au NP@MoS_2_ QDs, Au NPs (100 μM, elemental gold concentration), or MoS_2_ QDs (100 μM), or in RPMI-1640 medium (10% FBS) at 37 °C for 12 h. Afterwards the cells were rinsed with PBS five times to wash away excess nanoparticles for the Raman test. The measurements were performed directly on individual cells per group in the PBS buffer using a Renishaw inVia confocal Raman microspectrometer with a 785-nm excitation laser. A 50× objective lens with a ~1 μm spot size was applied to focus the laser beam. The SERS spectra were acquired with a 4 s integration time in static mode at a wavenumber center of 1200 cm^−1^. The Raman mapping was carried out under the streamline mode with a 2.45 s exposure time over a wavenumber range of 600–1750 cm^−1^. Each experiment was conducted at least five times.

## 3. Results and Discussion

### 3.1. Characterizations of Core@Shell Structure Au NP@MoS_2_ QDs 

**Figure 1:**
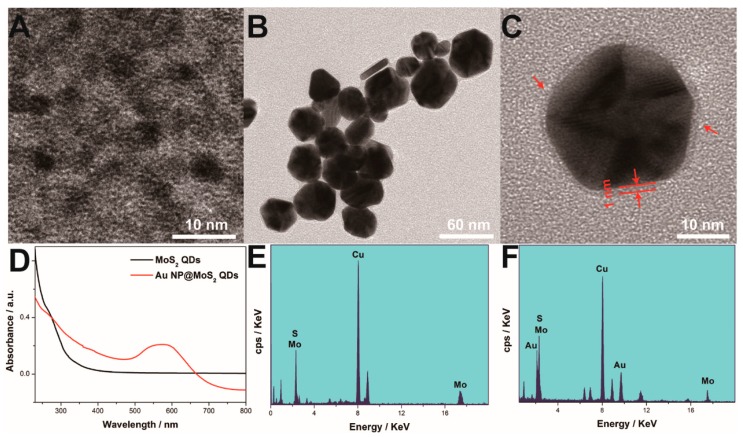
TEM images of MoS_2_ QDs (**A**) and Au NP@ MoS_2_ QDs at (**B**) low magnification as well as (**C**) high magnification (The red arrows pointed to the shells of the hybrids). UV-Vis spectra of MoS_2_ QDs and Au NP@ MoS_2_ QDs (**D**). EDX spectra of MoS_2_ QDs (**E**) and Au NP@ MoS_2_ QDs (**F**).

The morphologies of MoS_2_ QDs were characterized by TEM analysis (Figure 1A). The sizes of the MoS_2_ QDs are in the range of about 2–5 nm (Figure S1A). Figure 1B clearly displays the TEM images of the Au NP@MoS_2_ QDs hybrids with nearly spherical shape prepared via a simple method where the mixture of MoS_2_ QDs and HAuCl_4_ was heated to 80 °C for 10 min, and the mean diameter of the hybrids is ca. 43 nm (Figure S1B). The color of the mixture changed from light yellow to amaranth which demonstrated the formation of Au NPs. During the synthesis, MoS_2_ QDs were used to directly reduce the Au ions to gold NPs by the formation of the ${\mathrm{MoS}}_{2}/{\mathrm{AuCl}}_{4}^{-}$ redox pair, consistent with the previous study [37]. At higher magnification in Figure 1C, the core@shell structure of the nanocomposites with the ultrathin MoS_2_ QDs-coating (pointed by the red arrows) of ca. 1 nm can be seen. The UV-Vis spectrum of the MoS_2_ QDs (Figure 1D) illustrates a broad absorption band at around 270 nm which is assigned to the excitonic features of the MoS_2_ QDs [38]. After reacting with HAuCl_4_, a new absorption band consistent with the Au plasmon band at 578 nm appears, suggesting the formation of Au NPs. Meanwhile, along with the existence of the absorption peak at around 270 nm in the red line, it can be deduced that the integration of Au NP and MoS_2_ QDs occurs. The EDX spectra exhibit the presence of Mo and S elements in the MoS_2_ QDs (Figure 1E) and Mo, S, and Au elements in the nanocomposites (Figure 1F), which further demonstrate the generation of the Au NP@MoS_2_ QDs hybrids.

### 3.2. The SERS Activities of Au NP@MoS_2_ QDs Nanocomposites

**Figure 2:**
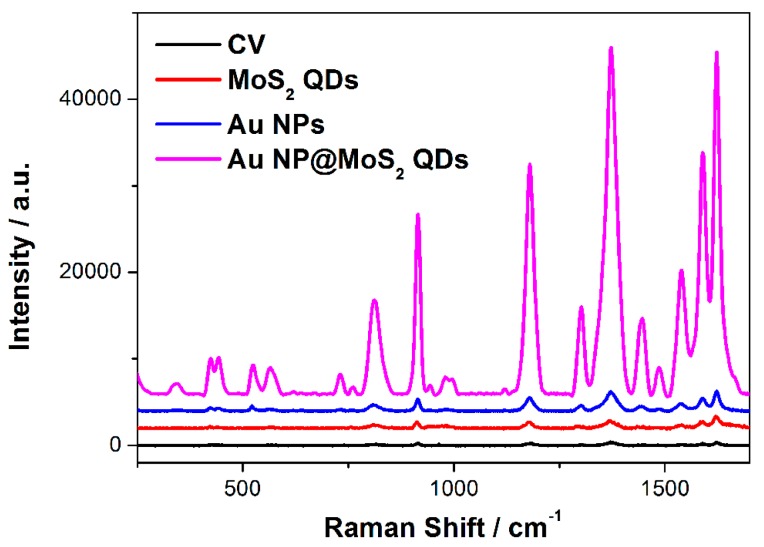
SERS spectra of 5 μM CV in Au NP@MoS_2_ QDs, MoS_2_ QDs, and Au NPs aqueous solution, and the normal Raman spectrum of the 1 mM CV aqueous solution.

In order to investigate the SERS performance of the Au NP@MoS_2_ QDs, herein, we employed crystal violet (CV) as the probe molecule due to its well-established and distinct Raman features. As shown in Figure 2, the SERS signal of 5 μM CV with Au NP@MoS_2_ QDs as the substrate is sharply enhanced. The prominent characteristic peaks of CV at 1539, 1588, and 1620 cm^−1^ are associated with the ring C–C stretching mode; the band at 1370, 1178, and 916 cm^−1^ are attributed to the N-phenyl stretching vibration, C–H bending mode, and in-plane phenyl deformation mode, respectively. In contrast, the SERS intensity of CV induced by Au NPs or MoS_2_ QDs is much weaker than that by Au NP@MoS_2_ QDs. The reasons for the superior SERS performance of the core@shell nanohybrids are as follows. First, as the enhanced signals of CV on Au NPs are mainly attributed to the electromagnetic enhancement and as MoS_2_ QDs can provide chemical enhancement, the hybrids combining both of these advantages show a higher SERS performance. Second, the MoS_2_ QDs can adsorb multifarious target molecules effectively. Figure S2 shows the UV-Vis spectra of the 1 mM CV solution and the solution after it was incubated with Au NPs or the hybrids. Obviously, the absorbance intensity of the CV solution after the absorption by Au NPs is much higher than that of the solution after absorption by the MoS_2_ QDs wrapped Au NPs. Third, the hybrids can be a chosen semiconductor system to form a molecule-semiconductor system with the CV molecules (with resonance at 576 nm, Figure S2), because the hybrids have a plasmon resonance at 578 nm near the CV molecular resonance. The combined system benefiting from the coupling effect of multiple resonances (surface plasmon, exciton, and charge-transfer) can lead to the strong SERS effect [30]. Finally, the MoS_2_ QDs shell-isolated substrate can free bare Au NPs from the harsh chemical environment and various metal-molecule interactions, leading to the high SERS detection sensitivity. 

For quantification, the enhancement factor (EF) was estimated using the following equation:

$$\mathrm{EF}=\frac{I_{SERS}}{C_{SERS}}\times \frac{C_{Raman}}{I_{Raman}}$$

where *I_SERS_* and *I_Raman_* represent the band intensities of the SERS spectrum and the normal Raman spectrum, respectively; and *C_SERS_* and *C_Raman_* are the concentrations of the probe molecules used for SERS and normal Raman measurements, respectively. According to the CV characteristic band at 1620 cm^−1^, the EF of Au NP@MoS_2_ QDs is calculated to be 3.6 × 10^4^ and is about 18 times stronger than that of the Au NPs (EF ≈ 2 × 10^3^) and 30 times stronger than that of the MoS_2_ QDs (EF ≈ 1.2 × 10^3^). This result indicates the good SERS effect of the hybrid as the substrate.

**Figure 3:**
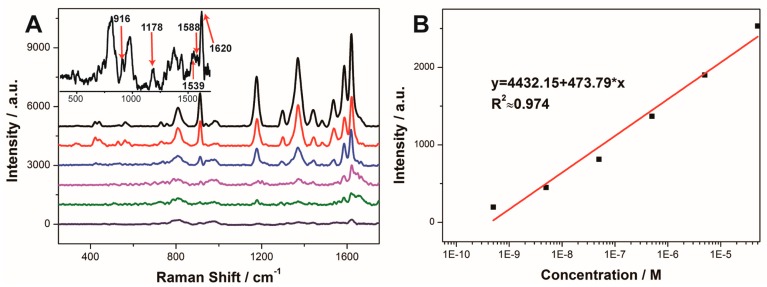
SERS spectra of CV with different concentrations in 200 μM Au NP@MoS_2_ QDs aqueous solution (**A**) and the corresponding quantitative curves (using the band at 1620 cm^−1^, **B**). Insert: SERS spectrum of 0.5 nM CV in the 200 μM nanocomposite suspension.

To further evaluate the SERS performance of the substrate, SERS spectra of the Au NP@MoS_2_ QDs suspension with the addition of different concentrations of CV are shown in Figure 3A. It is apparent that the SERS intensity decreases with the decline of the CV concentration along with the absence of some intrinsic bands. When the concentration of CV decreases to 0.5 nm, the bands at 804, 916, 1178, 1539, 1588, and 1620 cm^−1^ are still apparent (seen the insert of Figure 3A). The integrated intensity of the SERS band at 1620 cm^−1^ is used for quantification. As plotted in Figure 3B, the linear response from 0.5 nM to 50 μM of CV with the reasonable relationship is defined as y = 473.79x + 4432.15 (R^2^ ≈ 0.974). In this case, the limit of detection (LOD) is 0.5 nM, exhibiting the excellent SERS activity.

**Figure 4:**
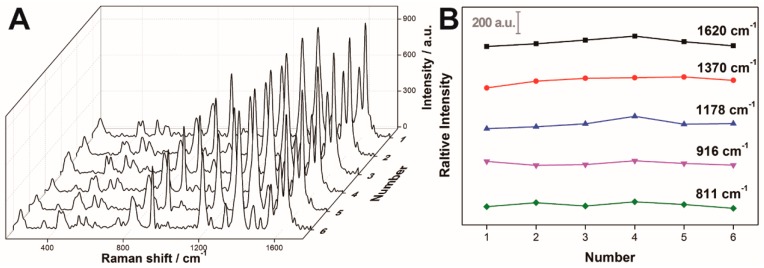
SERS spectra of CV induced by Au NPs@MoS_2_ QDs at six randomly selected positions (**A**); Uniformities of the SERS intensities of five characteristic Raman bands on the basis of the SERS detection of the six samples (**B**).

In practical applications, it is vital to accomplish uniformity of the SERS-active substrate. Six different positions on the sample are randomly chosen for SERS measurement, and each position was tested three times. In Figure 4A, the SERS spectrum of each sample has uniform features with very minimal variations in their signal intensities, which indicates the acceptable reproducibility of the Au NPs@MoS_2_ QDs substrate. For the purpose of further assessment of the hybrids, the SERS intensity of five prominent bands at 811, 916, 1178, 1370, and 1620 cm^−1^ were collected to calculate the relative standard deviations (RSDs) with the values of 3.6%, 3.8%, 6.2%, 3.9%, and 8.11% for the statistically meaningful analysis (Figure 4B). These RSD values are within the acceptable standard (less than 20%) [39]. The main reason for the satisfactory reproducibility of the as-prepared hybrids may be attributed to the chemically inert MoS_2_ QDs-shell that isolates the Au NP from the analyte molecules, which reduces the disturbed signals and enables the SERS analysis with more well-defined molecular interactions.

**Figure 5:**
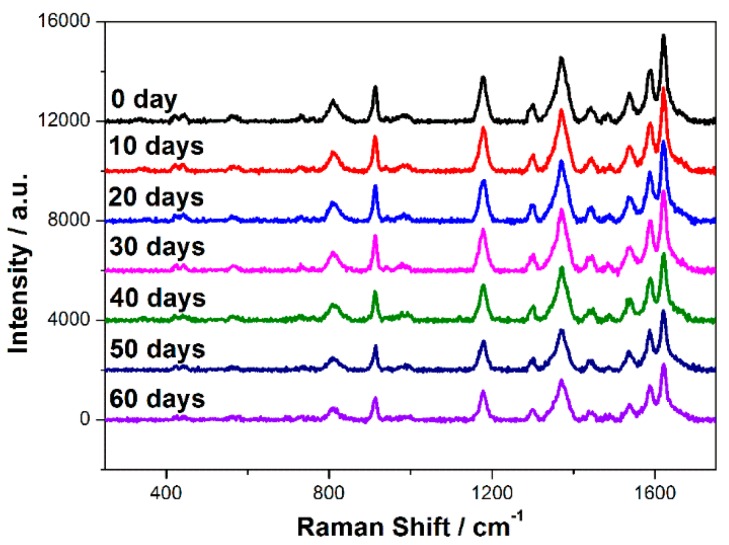
The SERS stability of Au NPs@MoS_2_ QDs.

Besides the sensitivity and the reproducibility, the stability of the substrate is also estimated by conducting the SERS experiment on the fresh sample of 5 μM CV in Au NPs@MoS_2_ QDs suspension every ten days as the substrate is stored at 4 °C with the treatment of light avoidance in the refrigerator. In Figure 5, it is noted that the average SERS intensity at 1370 cm^−1^ of the CV shows a degree of decay of about 36.6% after 60 days, offering an acceptable stability of the SERS substrate. In contrast, for the Au NPs system, the signals from CV show more than a 63.4% decrease relative to the initial response (Figure S3). Thus, Au NPs@MoS_2_ QDs, taking advantages of high SERS activity, adequate reproducibility, and satisfactory stability, may be a promising candidate for applications in pollutant detection and biological systems.

### 3.3. Cellular SERS Measurement and Label-Free Bio-Imaging

**Figure 6:**
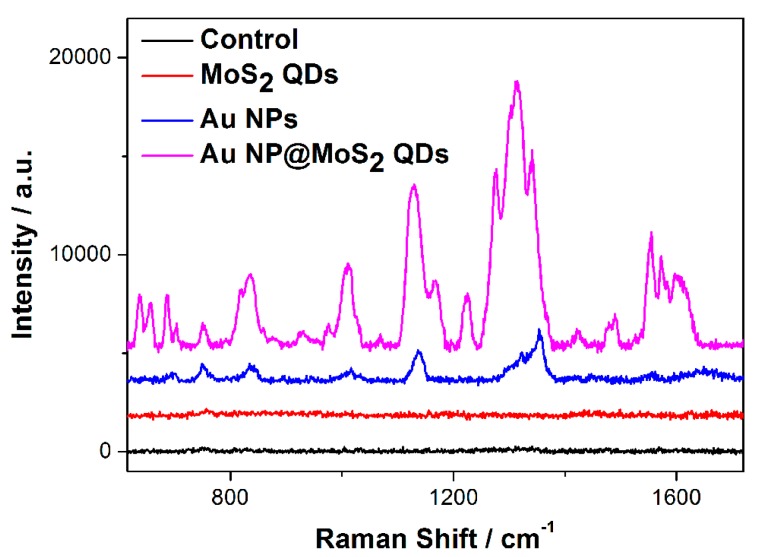
The mean SERS spectra of 4T1 cells triggered by MoS_2_ QDs, Au NPs, and the nanohybrids after incubation of 12 h.

With the discovery of the prominent SERS features and the great biocompatibility of the MoS_2_ QDs [28,40,41], we further investigate the capability of the Au NP@MoS_2_ QDs core@shell nanostructures for biomedicine applications. 4T1 cells treated with the nanohybrids or Au NPs for 12 h were used for Raman scanning under the confocal Raman microspectrometer with laser excitation at 785 nm. Figure 6 demonstrates the mean vibrational spectra of 40 SERS spectra of 4T1 cells with or without each label-free SERS probe. The SERS spectra were obtained in static mode with a wavenumber center at 1200 cm^−1^. No Raman signal is observed in the cells without nanoparticles or with MoS2 QDs under our experimental conditions. After incubation of the Au NPs for 12 h, only a small number of the Raman peaks appeared from the vibration of the cellular compositions, such as 749 cm^−1^, 835 cm^−1^, 1016 cm^−1^, 1136 cm^−1^, 1320 cm^−1^, 1353 cm^−1^, 1556 cm^−1^, and 1646 cm^−1^. Furthermore, the SERS intensities are relatively weak (Table S1). Although the hybrid-induced mean SERS spectrum of 4T1 cells had a similar spectral pattern when compared with that of the Au NPs, more intense SERS signals were acquired and more abundant SERS peaks emerged. The SERS spectrum caused by the Au NP@MoS_2_ QDs system shows intense vibration bands at 835 cm^−1^ (ring breathing mode of tyrosine or asymmetric O–P–O stretching vibration of DNA/RNA), 1010 cm^−1^ (symmetric ring breathing mode of phenylalanine), 1130 cm^−1^ (C–N stretching mode of protein, chain C–C stretching of lipid, or C–O/C–C in disaccharide) and 1600 cm^−1^ (phenylalanine or tyrosine). More distinct SERS bands were associated with the protein at 655 cm^−1^ (C–C twisting in protein), 976 cm^−1^ (tryptophan or ring deformation in tyrosine), 1224 cm^−1^ (random coils Amide III), 1554 cm^−1^ (Amide II), 1572 cm^−1^ (Amide II and tryptophan), etc. These data indicate that the Au NP@MoS_2_ QDs can be used as a SERS probe in the complex cellular environment. Detailed assignments are summarized in Table S2. 

**Figure 7:**
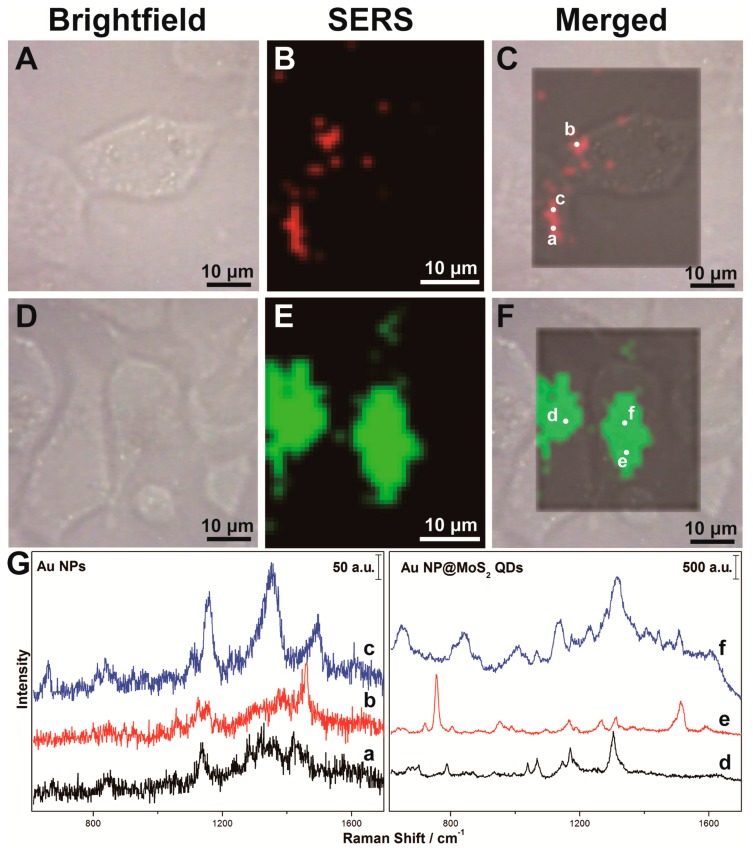
The label-free NIR SERS imaging and detection of 4T1 cells. The images are taken after incubation of Au NPs (**A**–**C**) and Au NPs@MoS2 QDs (**D**–**F**). (**G**) The raw SERS spectral lines of different spots randomly marked in the imaging of **C** and **F**.

The as-synthesized Au NP@MoS_2_ QDs were further used for label-free SERS mapping with the integrated intensity from 1200 to 1700 cm^−1^. Figure 7A–C shows an ambiguous and heterogeneous signal distribution because of the weak vibrational signals of the components in cells induced by Au NPs. In contrast, the 4T1 cells under the existence of Au NP@MoS_2_ QDs reveal a clear SERS image with a defined outline which is well in accordance with the bright-field microscopic image (Figure 7D–F). The overlap images of the SERS and bright-field images suggest that the endocytosed nanostructures are situated almost in the cytoplasm. The Raman features of the spots that were randomly marked in the SERS images (Figure 7C,F) have been plotted in Figure 7G. These raw spectral lines from the 4T1 cells caused by the SERS probes corresponding to the mean spectra in Figure 7G further demonstrate the much more effective SERS activity of the Au NP@MoS_2_ QDs with a better signal-to-noise ratio. The spectral fluctuations of the six SERS lines also reveal a heterogeneous cellular composition in the cytoplasmic areas. In addition, the plentiful cellular SERS bands of the SERS spectral lines originating from a 4T1 cell containing the hybrids (the right part of Figure 7G) with inevitable autofluorescence background are observed without any interference, which demonstrates the superiority of the Au NP@MoS_2_ QDs as SERS active probes for cellular SERS analysis. It is worth noting that the Raman signatures of the cellular components can be prone to being drowned out in the inherent Raman features of graphene and its derivatives when they are selected as the SERS substrates for cell analysis [24,42]. Hence, MoS_2_ QDs may be the better alternative due to their intrinsic vibrational bands in the Raman-silence region of cells. 

## 4. Conclusions

Au NP@MoS_2_ QDs with an ultrathin MoS_2_ QDs shell of ca. 1 nm have been successfully prepared by a rapid reduction of the Au precursor with MoS_2_ QDs. The nanocomposites show high SERS sensitivity for the detection of CV with a LOD of 0.5 nM and are much more competitive than pure Au NPs and MoS_2_ QDs, which is mainly due to the additional chemical enhancement mostly caused by the MoS_2_ QDs and great adsorbability of the MoS_2_ QDs. Furthermore, the SERS reproducibility experiment has a satisfactory result, as the RSDs of the main Raman bands’ intensities are less than 8.11%, and the SERS stability of the nanocomposites has been proven. We have revealed the capability of Au NP@MoS_2_ QDs to be an ultrasensitive SERS probe for cell analysis and label-free SERS imaging. In conclusion, Au NP@MoS_2_ QDs have a promising future as a high performance SERS-active substrate for pollutants or toxicant detection associated with our daily life and biological analysis.

## Supplementary Materials

The following are available online at www.mdpi.com/1996-1944/10/6/650/s1, Figure S1: The mean diameter of MoS_2_ QDs (A) and Au NP@ MoS_2_ QDs (B), respectively, Figure S2: UV-Vis spectra of a 1 mM CV solution, and the remaining solution of 1 mM CV after the adsorption by 1 mM Au NPs or Au NP@MoS_2_ QDs, Figure S3: The stability of Au NPs, Table S1: The assignments of the Raman bands of 4T1 cells incubated with AuNPs, Table S2: The assignments of the Raman bands of 4T1 cells incubated with AuNP@MoS_2_ QDs.

## References

[1] Chen Y., Tan C.L., Zhang H., Wang L.Z. (2015). Two-dimensional graphene analogues for biomedical applications. Chem. Soc. Rev..

[2] Liu T., Cheng L., Liu Z. (2015). Two Dimensional Transitional Metal Dichalcogenides for Biomedical Applications. Acta Chim. Sin..

[3] Mak K.F., He K.L., Shan J., Heinz T.F. (2012). Control of valley polarization in monolayer MoS_2_ by optical helicity. Nat. Nanotechnol..

[4] Zaumseil J. (2014). Electronic Control of Circularly Polarized Light Emission. Science.

[5] Wang Q.H., Kalantar-Zadeh K., Kis A., Coleman J.N., Strano M.S. (2012). Electronics and optoelectronics of two-dimensional transition metal dichalcogenides. Nat. Nanotechnol..

[6] Gao W., Wang M., Ran C., Li L. (2015). Facile one-pot synthesis of MoS_2_ quantum dots-graphene-TiO_2_ composites for highly enhanced photocatalytic properties. Chem. Commun..

[7] Qiao W., Yan S., Song X., Zhang X., Sun Y., Chen X., Zhong W., Du Y. (2015). Monolayer MoS_2_ quantum dots as catalysts for efficient hydrogen evolution. RSC Adv..

[8] Tan Y., He R., Cheng C., Wang D., Chen Y., Chen F. (2014). Polarization-dependent optical absorption of MoS_2_ for refractive index sensing. Sci. Rep..

[9] Ganatra R., Zhang Q. (2014). Few-Layer MoS_2_: A Promising Layered Semiconductor. ACS Nano.

[10] Bhanu U., Islam M.R., Tetard L., Khondaker S.I. (2014). Photoluminescence quenching in gold—MoS_2_ hybrid nanoflakes. Sci. Rep..

[11] Lee H.S., Min S.-W., Chang Y.-G., Park M.K., Nam T., Kim H., Kim J.H., Ryu S., Im S. (2012). MoS_2_ Nanosheet Phototransistors with Thickness-Modulated Optical Energy Gap. Nano Lett..

[12] Arul N.S., Nithya V.D. (2016). Molybdenum disulfide quantum dots: Synthesis and applications. RSC Adv..

[13] Ren X., Pang L., Zhang Y., Ren X., Fan H., Liu S. (2015). One-step hydrothermal synthesis of monolayer MoS_2_ quantum dots for highly efficient electrocatalytic hydrogen evolution. J. Mater. Chem. A.

[14] Xu S., Li D., Wu P. (2015). One-Pot, Facile, and Versatile Synthesis of Monolayer MoS_2_/WS_2_ Quantum Dots as Bioimaging Probes and Efficient Electrocatalysts for Hydrogen Evolution Reaction. Adv. Funct. Mater..

[15] Dong H., Tang S., Hao Y., Yu H., Dai W., Zhao G., Cao Y., Lu H., Zhang X., Ju H. (2016). Fluorescent MoS_2_ Quantum Dots: Ultrasonic Preparation, Up-Conversion and Down-Conversion Bioimaging, and Photodynamic Therapy. ACS Appl. Mater. Interfaces.

[16] Lin H., Wang C., Wu J., Xu Z., Huang Y., Zhang C. (2015). Colloidal synthesis of MoS_2_ quantum dots: Size-dependent tunable photoluminescence and bioimaging. New J. Chem..

[17] Gu W., Yan Y., Cao X., Zhang C., Ding C., Xian Y. (2016). A facile and one-step ethanol-thermal synthesis of MoS_2_ quantum dots for two-photon fluorescence imaging. J. Mater. Chem. B.

[18] Jin X., Fan X., Tian J., Cheng R., Li M., Zhang L. (2016). MoS_2_ quantum dot decorated g-C_3_N_4_ composite photocatalyst with enhanced hydrogen evolution performance. RSC Adv..

[19] Gan Z., Gui Q., Shan Y., Pan P., Zhang N., Zhang L. (2016). Photoluminescence of MoS_2_ quantum dots quenched by hydrogen peroxide: A fluorescent sensor for hydrogen peroxide. J. Appl. Phys..

[20] Qian X.M., Nie S.M. (2008). Single-molecule and single-nanoparticle SERS: From fundamental mechanisms to biomedical applications. Chem. Soc. Rev..

[21] Otto A., Mrozek I., Grabhorn H., Akemann W. (1992). Surface-enhanced Raman scattering. J. Phys..

[22] Ko H., Singamaneni S., Tsukruk V.V. (2008). Nanostructured Surfaces and Assemblies as SERS Media. Small.

[23] Luo P., Li C., Shi G. (2012). Synthesis of gold@carbon dots composite nanoparticles for surface enhanced Raman scattering. Phys. Chem. Chem. Phys..

[24] Liu Z., Hu C., Li S., Zhang W., Guo Z. (2012). Rapid intracellular growth of gold nanostructures assisted by functionalized graphene oxide and its application for surface-enhanced Raman spectroscopy. Anal. Chem..

[25] Xu S., Jiang S., Wang J., Wei J., Yue W., Ma Y. (2016). Graphene isolated Au nanoparticle arrays with high reproducibility for high-performance surface-enhanced Raman scattering. Sens. Actuators B.

[26] Ge J., Li Y., Wang J., Pu Y., Xue W., Liu X. (2016). Green synthesis of graphene quantum dots and silver nanoparticles compounds with excellent surface enhanced Raman scattering performance. J. Alloys Compd..

[27] Ling X., Fang W., Lee Y.H., Araujo P.T., Zhang X., Rodriguez-Nieva J.F., Lin Y., Zhang J., Kong J., Dresselhaus M.S. (2014). Raman enhancement effect on two-dimensional layered materials: Graphene, h-BN and MoS_2_. Nano Lett..

[28] Muehlethaler C., Considine C.R., Menon V., Lin W.-C., Lee Y.-H., Lombardi J.R. (2016). Ultrahigh Raman Enhancement on Monolayer MoS_2_. ACS Photonics.

[29] Najmaei S., Mlayah A., Arbouet A., Girard C., Leotin J., Lou J. (2014). Plasmonic Pumping of Excitonic Photoluminescence in Hybrid MoS_2_-Au Nanostructures. ACS Nano.

[30] Lombardi J.R., Birke R.L. (2014). Theory of Surface-Enhanced Raman Scattering in Semiconductors. J. Phys. Chem. C.

[31] Su S., Zhang C., Yuwen L., Chao J., Zuo X., Liu X., Song C., Fan C., Wang L. (2014). Creating SERS hot spots on MoS_2_ nanosheets with in situ grown gold nanoparticles. ACS Appl. Mater. Interfaces.

[32] Wu J.-Y., Zhang X.-Y., Ma X.-D., Qiu Y.-P., Zhang T. (2015). High quantum-yield luminescent MoS_2_ quantum dots with variable light emission created via direct ultrasonic exfoliation of MoS_2_ nanosheets. RSC Adv..

[33] Liu T., Chao Y., Gao M., Liang C., Chen Q., Song G., Cheng L., Liu Z. (2016). Ultra-small MoS_2_ nanodots with rapid body clearance for photothermal cancer therapy. Nano Res..

[34] Li J.F., Anema J.R., Wandlowski T., Tian Z.Q. (2015). Dielectric shell isolated and graphene shell isolated nanoparticle enhanced Raman spectroscopies and their applications. Chem. Soc. Rev..

[35] Li J.F., Tian X.D., Li S.B., Anema J.R., Yang Z.L., Ding Y., Wu Y.F., Zeng Y.M., Chen Q.Z., Ren B. (2013). Surface analysis using shell-isolated nanoparticle-enhanced Raman spectroscopy. Nat. Protoc..

[36] Li J.F., Huang Y.F., Ding Y., Yang Z.L., Li S.B., Zhou X.S., Fan F.R., Zhang W., Zhou Z.Y., Wu D.Y. (2010). Shell-isolated nanoparticle-enhanced Raman spectroscopy. Nature.

[37] Kim J., Byun S., Smith A.J., Yu J., Huang J. (2013). Enhanced Electrocatalytic Properties of Transition-Metal Dichalcogenides Sheets by Spontaneous Gold Nanoparticle Decoration. J. Phys. Chem. Lett..

[38] Chikan V., Kelley D.F. (2002). Size-dependent spectroscopy of MoS_2_ nanoclusters. J. Phys. Chem. B.

[39] Dinish U.S., Yaw F.C., Agarwal A., Olivo M. (2011). Development of highly reproducible nanogap SERS substrates: Comparative performance analysis and its application for glucose sensing. Biosens. Bioelectron..

[40] Teo W.Z., Chng E.L.K., Sofer Z., Pumera M. (2014). Cytotoxicity of Exfoliated Transition-Metal Dichalcogenides (MoS_2_, WS_2_, and WSe_2_) is Lower than That of Graphene and its Analogues. Chemistry.

[41] Zhang X.D., Zhang J., Wang J., Yang J., Chen J., Shen X., Deng J., Deng D., Long W., Sun Y.M. (2016). Highly Catalytic Nanodots with Renal Clearance for Radiation Protection. ACS Nano.

[42] Dresselhaus M.S., Jorio A., Hofmann M., Dresselhaus G., Saito R. (2010). Perspectives on Carbon Nanotubes and Graphene Raman Spectroscopy. Nano Lett..

