# Supplementary material for: Synthesis of Au NP@MoS2 Quantum Dots Core@Shell Nanocomposites for SERS Bio-Analysis and Label-Free Bio-Imaging
Source: Materials (Basel). 2017 Jun 13;10(6):650. doi: 10.3390/ma10060650 (PMC5554031; doi:10.3390/ma10060650)
Supplement: Supplementary file 1 [file materials-10-00650-s001.pdf]

Supplementary Materials: Synthesis of Au NP@MoS_2 Quantum Dots Core@Shell Nanocomposites for SERS Bio-Analysis and Label-Free Bio-Imaging

Xixi Fei , Zhiming Liu, Yuqing Hou, Yi Li, Guangcun Yang, Chengkang Su, Zhen Wang, Huiqing Zhong, Zhengfei Zhuang and Zhouyi Guo

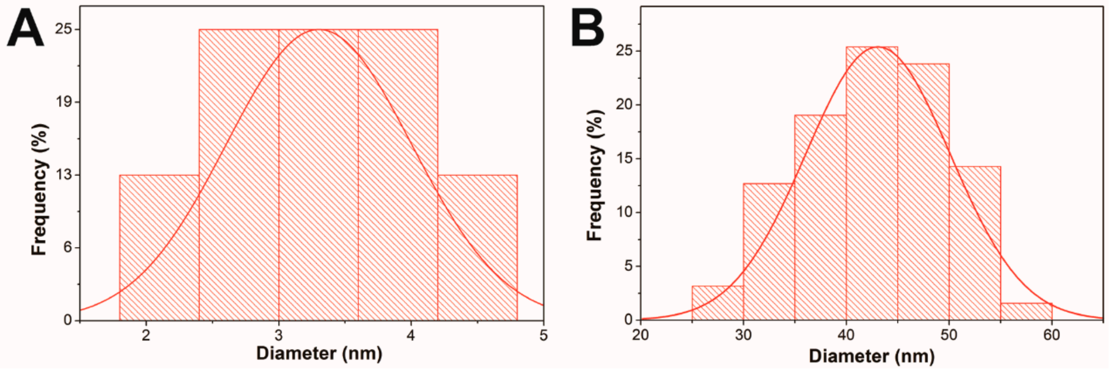

Figure S1. The mean diameter of MoS_2 QDs (**A**) and Au NP@ MoS_2 QDs (**B**), respectively.

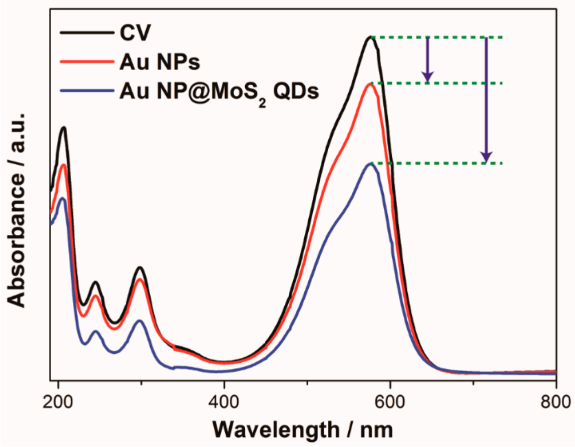

Figure S2. UV-Vis spectra of a 1 mM CV solution, and the remaining solution of 1 mM CV after the adsorption by 1 mM Au NPs or Au NP@MoS_2 QDs.

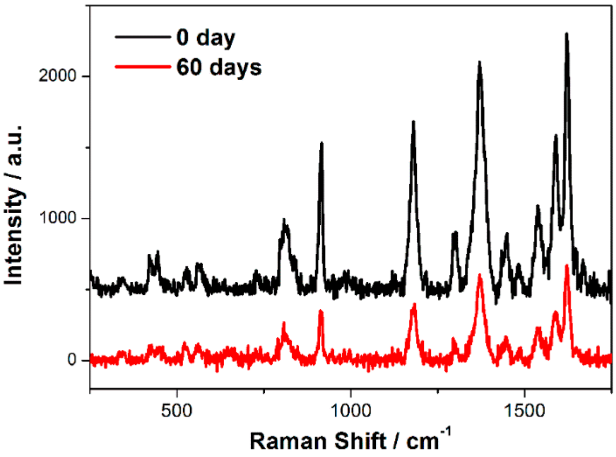

Figure S3. The stability of Au NPs.

Table S1. The assignments of the Raman bands of 4T1 cells incubated with AuNPs.

Raman Shift (cm^{-1}) 4T1	Tentative assignment
749	T (d); Trp ring breath (p)
835	Tyr ring breath (p); O-P-O asym str (d)
1008	Phe ring sym breath (p)
1136	Proline(p)
1320	G(d), CH def (p)
1353	CH2/CH3 wag (p)
1556	Amide II(p)
1646	Amide I(p)

Abbreviations: p, protein; l, lipid; d, DNA/RNA; Tyr, tyrosine; Trp, Tryptophan; T, thymine; G, guanine; str, stretching; breath, breathing; wag, wagging; sym, symmetrical; asym, asymmetrical; bk, backbone.

Table S2. The assignments of the Raman bands of 4T1 cells incubated with AuNP@MoS_2 QDs.

Raman Shift (cm^{-1}) 4T1	Tentative assignment
636	Tyr, COO- bend or C-S str (p)
655	C-C twist Tyr (p)
685	G(d)
703	Cholesterol (l)
749	T (d); Trp ring breath (p)
820	Try ring breath (p)
835	Tyr ring breath (p); O-P-O asym str (d)
930	C-C bk str α helix (p); C-O-C glycos (c)
976	Trp, Tyr ring def (p)
1010	Phe ring sym breath (p)
1069	C-N str (p); Chain C-C str (l)
1130	C-N str (p); Chain C-C str (l); Disaccharide: C-O/C-C (c)
1168	C-C/C-N str (p)
1224	Amide III random coils (p)
1276	Amide III α helix (p), CH2/CH3 def (p, l)
1316	G(d), CH def (p)
1340	CH_2/CH_3 twist, CH_2/CH_3 wag (p)
1353	CH2/CH3 wag (p)
1420	CH3 asym str (p, l)
1479	G, A (d)
1489	A(d)
1554	Amide II (p)
1572	Amide II, Trp (p)
1600	Phe, Tyr (p)

Abbreviations: p, protein; l, lipid; d, DNA/RNA; c, carbohydrate; Tyr, tyrosine; Trp, Tryptophan; Phe, phenylalanine; A, adenine; T, thymine; G, guanine; str, stretching; def, deformation; twist, twisting; breath, breathing; wag, wagging; sym, symmetrical; asym, asymmetrical; bk, backbone.
